# The development of a scheme of Public & Patient Involvement (PPI) in the Wales Cancer Research Centre (WCRC) and reflections on the process

**DOI:** 10.1186/s40900-020-00221-6

**Published:** 2020-09-21

**Authors:** Jim Fitzgibbon, Kate Cleary, Annmarie Nelson

**Affiliations:** 1 Lead Research Partner, Wales Cancer Research Centre, Cardiff, Wales UK; 2 Administrative Officer, Wales Cancer Research Centre, Cardiff, Wales UK; 3 Lead Researcher for Public and Patient Involvement, Wales Cancer Research Centre and Marie Curie Professor of Supportive and Palliative Care, Marie Curie Palliative Care Research Centre, Cardiff, Wales UK

**Keywords:** Research partner, Wales Cancer Research Centre, Public and Patient Involvement

## Abstract

The Wales Cancer Research Centre (WCRC) was established in 2015. It made an early and strong commitment to Public and Patient Involvement (PPI) in all its work. That commitment was made manifest through the immediate appointment of Lay and Researcher Leads and an administrator to develop and implement a scheme of PPI.

At the core of the scheme was the allocation to each of the centre’s four themes two Research Partners (RPs), who were to offer routine and strategic support to researchers but also to have a wider ambassadorial role, acting as champions for PPI.

The RPs were appointed through a full recruitment process and supported financially, with a ‘budget’ of 10 half days per annum, with training where needed and supported by a mentor. Their core tasks were defined through an audit of then current practice in PPI within the themes. Monitoring of progress was undertaken at regular group PPI meetings, reports to the centre’s funders against key performance indicators and against a rerun of the initial audit.

A library of documents was produced to support this work, including a centre policy statement, procedures for the recruitment, training and support of RPs, a partnership agreement between RPs and researchers and a mentorship agreement. Most recently procedures have been drafted to assess the impact on research of PPI.

The scheme has been regarded as largely successful by researchers, RPs and the Centre’s External Advisory Board.

However there remains much to do to ensure consistently high quality involvement of RPs in the centre’s research. A significant stumbling block to making progress has been the lack of time given to researchers by funders to become involved in PPI. A reflection on progress against the UK Standards for PPI has identified a number of key actions for the future. They include the roll out of a scheme to assess the impact of PPI and to increase diversity in the centre’s pool of RPs.

## Plain English summary

This article maps the foundation and development of a scheme of Public and Patient Involvement (PPI) in the Wales Cancer Research Centre (WCRC) over its first 5 years of existence: It also offers reflections on the success or otherwise of the endeavour.

It describes the very particular approach to PPI where public contributors, known as Research Partners (RPs), are defined as consultants to researchers who work together to achieve the centre’s objectives, supported by a range of protocols and other documents. Emphasis is given to the importance of leadership in carrying the scheme forward.

Finally there is some reflection on the experience against the UK Standards for PPI and suggestions of tasks which need to be taken forward in the centre’s next 5 years, not least of all the roll out of the scheme to assess the impact of PPI and a programme to promote diversity in the centre’s pool of RPs.

## Background

The involvement of members of the public in research has been a condition of most funders of health research for a number of years [1, 2, 5, 6]. However, it is apparent from the UK Clinical Research Collaboration’s recent survey that practice in this field remains uneven [4]. Barriers to involvement include lack of time for researchers to support lay representatives, a dearth of training for all parties, inadequate funding and challenges in the recruitment of the public to trial management groups [3, 7, 9]. There also remains doubt in some minds that public involvement has any impact, or adds value to, the quality of research [8]. This latter issue remains hotly contested. There have been a number of attempts to measure impact and some positive endorsements of lay involvement in research, but some researchers remain sceptical of its value.

Most recently the publication of the UK Standards for Public Involvement in Research has raised the profile of public involvement and helped to clarify what needs to be done to achieve high quality in its implementation and the measurement of its impact [10]. The Standards are not, however, prescriptive, allowing local solutions to their implementation. It may be worth adding that they raise another key issue alongside impact assessment which has not been adequately addressed in recent history, that of the lack of diversity in the cadre of members of the public involved in research.

### The establishment of the centre, its vision, aims, approach and objectives

Launched in 2015, the WCRC is funded by the Welsh Government and is a part of Health and Care Research Wales’ infrastructure. It is led from Cardiff University, but with an all-Wales brief. It works alongside numerous partners with the cancer community including NHS Wales, other Welsh universities, cancer charities and the pharmaceuticals industry. It has a 5 year budget of approaching £5 m. The budget for PPI was a ‘guesstimate’ of need which has proven to be sufficient and flexible enough to meet additional needs, such as care costs, when they were identified over the course of the contract.

### PPI: WCRC’s ‘Golden thread’, its vision for PPI

From the outset, there was a clear aim that PPI should be fully integrated into all that the WCRC does. At the launch of the centre the metaphor of a ‘golden thread’ was used to highlight how PPI would be woven into everything the centre did. Since then this expression has been constantly referenced to depict PPI’s central role in the warp and weft of the WCRC. The vision for Patient and Public Involvement in the WCRC is to deliver “an active partnership between the public, researchers and others, to develop cancer research in Wales to improve their health and well being”.

### The centre’s aims for PPI were to

- Work with Health and Care Research Wales and other key partners in the public and third sectors to bring together expertise, insight and experience in the field of public involvement.
- Identify and maximise the appropriate opportunities for public involvement in cancer research in Wales, avoiding tokenism and ensuring involvement happens wherever it can add value.
- Embed public involvement across all aspects of the WCRC (including WCRC work packages and their advisory and governance groups).
- Work with key partners to develop capacity and capability for public involvement in cancer research in Wales.
- Learn and share knowledge and experiences of best practice in public involvement.
- Enable the public to influence policy, practice and research priorities.

### The approach to PPI

The posts of Lay and Researcher leads for PPI were filled through a competitive recruitment process, the former from within the Welsh Government’s pool of Research Partners (known as Research Partners or RPs). The process for both posts included advertisement and interview, against role descriptions and person specifications, by the Head of Centre and his senior staff. The rubric for the recruitment support and provision of training for RPs, including the Lay Lead was set out in its Standard Operating Procedures (SOPs), summarised in Table 1. The two leads coproduced all the documentation for PPI for the centre and led on all other aspects of this work. Original versions of the documents summarised on Tables 1, 2, 3, 4, 5, 6 and 7 are included in the WCRC website. They are all currently being reviewed in preparation for the centre’s next phase.

**Table 1:** Standard Operating Procedures (SOPs) for Public and Patient Involvement

• Recruitment: advice on recruitment of RPs (emphasising the need for at least two to each trial or study, a model followed in the themes), advertisement, development of role and person specifications and interview questions. Includes templates and flow diagrams to assist with all parts of the process. • Finance: advice on payment of honoraria and travelling and other expenses, to be made in accordance within the Welsh Government’s and INVOLVE’s schemes. • Induction, Mentoring and Training: advice on the induction, training and mentoring of RPs, either using Welsh Government’s services or by bespoke arrangements identified at induction or review. Mentoring provided by the Researcher and Lay Leads.

**Table 2:** Audit tool for establishing a baseline to adhering to the National standards for public involvement in research

• Rubric/instructions for Theme Research Partners about how to use the tool, with whom and when • Definitions of terms used in the document • Basic data about numbers of studies/trials undertaken, numbers of public contributors involved and a summary of engagement activities • Standard by standard, a set of questions and prompts to be used by the Theme Research Partners to identify progress against them and gaps needing to be filled • Priorities for action changed over time as a result of audit.

**Table 3:** Wales Cancer Research Centre Policy for Patient and Public Involvement and Engagement

• Definitions and examples of involvement and engagement • Statement of intent by the centre to achieve high quality involvement and engagement across its work on a routine basis • The scope of the policy and the responsibilities of the various partners in the centre’s work • Details on how the policy will be implemented structurally, financially and culturally, all to be underpinned with a joint statement of commitment by all partners • A set of key performance indicators against which to judge progress in achieving the policy • Mechanism for its review

**Table 4:** Partnership Agreement

• Sets out the mutual expectations of public contributors and researchers in their work with the centre • These are summarised under the headings Training and Induction, Communication and Practice (respect, confidentiality, recognising achievement, supportive administrative arrangements and behaviours)

**Table 5:** Terms of Reference for PPI Group of the Centre

• Sets out how the group will function and its membership • Defines the role, responsibilities and ways in which the group will work • Lists the group’s membership and allows for co-option of advisers • Provides for an annual review of its functioning

**Table 6:** Mentorship Agreement

• Sets out the broad spectrum of mutual expectations of public contributors and their mentors • As a baseline it enumerates the roles of the public contributors overall, their broad objectives and the commitment they make to achieving them • Outlines the resources and support available to enable them to achieve their objectives • Lists in detail the relative roles of both the researcher and public contributor in relation to the domains of Training and Induction, Communication and Practice • The mentor and public contributor are expected to sign/countersign the agreement

**Table 7:** Impact tool

• Includes a protocol that highlights specific areas to consider. These areas include assessing the difference that PPI makes to the research question, design, processes, progress, outcomes, dissemination and implementation of research. • Also includes a standard operating procedure, setting out a step by step process of how to measure the impact of the involvement of members of the public in research.	

The Lay and Researcher Leads recruited eight theme RPs, using the same SOPs, to offer guidance and support to researchers in the centre’s four themes. Together with a representative of the Welsh Government’s Support Centre and centre administration, these ten appointees formed a quarterly PPI Advisory Group, chaired by the Lay Lead, which steered the development of its PPI policy and practice. This group’s work was supported by a core team of the Researcher and Lay Leads and the centre’s PPI administrator which met on a weekly basis. The core team managed its workplan using project management software.

The RPs were appointed initially for 12 months and then confirmed in post for the period of the centre’s contract, subject to annual reviews. Given the unusual nature of their role and the commitment requested they were difficult posts to fill. Over the 5 year period it was necessary to advertise for replacement on four occasions. It was difficult to attract interest from across Wales, partly at least because of the geography of the country. Achieving diversity in the group also proved difficult. This is one of the two key priorities to be addressed in the next quinquennium, alongside the assessment of the impact of RPs' contributions to the quality of research. The theme RP roles were unlike those traditionally undertaken by members of the public in cancer research. While Theme RPs do, on request, complete the traditional tasks of commenting on protocols and other documents, their prime role is strategic and ambassadorial, to effectively co-lead with researchers on all aspects of the development of PPI within each research theme.

The centre’s RPs started work on a scoping exercise of each research theme, designed to identify what the barriers and obstacles were to implementing good PPI, and where good practice was already in place. The contents of the centre’s latest audit tool used in this exercise is summarised in Table 2. Aggregating all the scoping reports, made it possible to tailor PPI support to each research area.

The centre’s PPI policy (summarised in Table 3) was the last document to be set down on paper. This ‘late scribing’ was a deliberate decision based upon a desire to have a policy statement grounded in practice.

The centre’s partnership agreement, a statement of reasonable, mutual expectations of researchers and their RPs is outlined in Table 4.

### Objectives

The WCRC aimed to build upon the progress already made by its partner organisations, particularly the Marie Curie Palliative Care Research Centre, and take this work further by development of a scheme of patient involvement which set out the following objectives:

- To work with the Health and Care Research Wales Support Centre and other partners for advice, support and recruitment of lay representatives.
- To appoint lay representatives to the governance and advisory boards of the proposed centre and WCRC research projects.
- To ensure that lay representatives and researchers received appropriate training and support, either internally or through engagement with the Health and Care Wales Support Centre training programmes.
- To develop close working with a range of third sector organisations, establishing processes to ensure that there is alignment of the centre’s research priorities with those of the general public.
- To identify in each constituent organisation a senior member of staff to champion the involvement of the public in its work.
- To make sure that all public involvement in WCRC was co-ordinated and day-to-day support was provided for lay representatives by a paid member of staff or a volunteer.
- To work with Health and Care Research Wales to communicate to the public, cancer and research communities the WCRC’s commitment to this work.
- To develop mechanisms to evaluate and disseminate the impact of public involvement.
- To establish realistic budgets to support this public involvement work.
- To confirm public involvement in the WCRC by working with partner organisations to develop a standard approach and disseminating best practice through agreeing standard operating procedures, guidance and supporting paperwork.

### Metrics

The centre set out to achieve its vision through a series of the measurable actions with associated Key Performance Indicators (KPIs) which have either been achieved, wholly (w) or partly (p):

- Provision of advice and support for recruitment of lay representatives to the WCRC (w)
- Appointment of lay representatives to governance structures, advisory boards and research projects (w)
- Establishing a PPI group (which is lay-led with a majority of members who are lay representatives) to coordinate both PPI policy and practice (Terms of reference outlined in Table 5) (w)
- Undertaking scoping and baseline data collection exercises across the four themes (w)
- Provide appropriate training and support for lay representatives and researchers (Mentorship agreement summarised in Table 6) (p)
- Establishing processes to ensure that there is alignment of the centre’s research priorities with those of the general public (p)
- Making sure that all public involvement in WCRC is co-ordinated and day-to-day support is provided for lay representatives (w)
- Working with Health and Care Research Wales to communicate with the public, cancer and research communities WCRC’s commitment to this work (w)
- Developing mechanisms to evaluate and disseminate the impact of public involvement (p) (Impact tool summarised in Table 7)

- Demonstrating support for public involvement in its work (w)
- Embedding public involvement in Wales’ cancer research(w)
- Championing the involvement of the public in its work (w)

The achievement of these KPIs and further action required in relation to them is returned to later under Reflection.

### Reflection

In November 2019, NIHR, the Chief Scientist Office, Scotland, Health & Care Research Wales and the public Health Agency, Northern Ireland published a set of UK Standards for Public Involvement in Research [10]. They described the standards as:

- A framework for what good public involvement in research looks like and are adaptable to different situations
- Designed to encourage reflection and learning, including where lessons have been learned when public involvement has failed to lead to expected outcomes
- A tool to help people and organisations identify what they are doing well, and what needs improving
- Intended to be used with any method, or approach to public involvement in research (p2)

They are not absolutes but are intended to support improvement to PPI over time. One of the suggested uses for them is to ‘assess the strengths and weaknesses of (their) involvement in research and identify improvements’ (p2).

The Standards were not written when the WCRC was established and could not, therefore, be used as a framework for the establishment of its PPI scheme. However, they were largely the product of consideration of best PPI practice in the UK over time, much of which informed the centre’s approach to its work. Particular influences on the centre were the prior work of the Marie Curie Palliative Care Research Centre and of the Health and Care Research Wales Support Centre.

However, looking forward, the Standards will be the touchstone against which the centre will assess its PPI, both internally and nationally. To support this process the following paragraphs:

- Consider where the centre has made progress over the last 5 years against each of the Standards in turn.
- Suggest areas where it will need to particularly focus its efforts over its next phase.

1. Inclusive Opportunities

**Progress**

- Centre Lead RP involved in formulation of the proposal to renew its funding as a standing member of the Senior Leaders Group (Director, three Assistant Directors and senior administrator and Lead RP).

- All centre RPs offered honoraria for their inputs, travelling and subsistence allowances and for provision of childcare. Meetings take place at times and venues agreed with RPs

- Opportunities to become involved are all advertised widely through HCRW and its ‘community’ of RPs, as well as twitter and Facebook
- Opportunities can be undertaken in person or remotely via email and telephone or videoconferencing.

**Future action**

- Not all opportunities for individual trials or studies have inputs at the earliest stages. Centre RPs will continue to press for this to happen.
- Payment of honoraria is not always available for trials and studies prior to them ‘achieving their funding’. Work is in train to establish with HCRW a bespoke system where Welsh Government can make this happen.
- The ‘community’ from which RPs are mainly drawn is not sufficiently representative of the broader population of Wales. Work is in hand to produce guidance for researchers, including the production of case studies of good practice, on how to attract and retain a wider group of RPs. This work builds upon the products of an INVOLVE working group to which the Lay Lead RP contributed.

2. Working Together

**Progress**

- The centre’s definition of PPI and all its documentation including its policy statement has been coproduced between researchers, RPs and administrators.
- The quarterly PPI Group meeting enables RPs to contribute to the development and continuing implementation of the centre’s policy for PPI.
- All the RPs, including the Lead RP, have role descriptions. The centre’s Partnership Agreement sets out mutual expectations for researchers and RPs in their respective roles.
- RPs have been involved in the development of an all Wales cancer research strategy together with the third sector.
- One of the theme RPs was a member of the working group which produced the UK Standards
- Recognition of RPs’ work is given at centre conferences, in its reports to Welsh Government and in its annual report.

**Future action**

- More work needs to be done to ensure that all trials and studies in any way connected to the centre follow its policy and practice.
- There needs to be more consideration given to the very wide variety of the centre’s research and any adjustments that may need to be made to its PPI support to reflect this. For example, the centre’s structure for its next funding round has led to discussion about whether the theme RP model is appropriate to all phases and types of research.
- The centre’s Partnership Agreement is being reviewed and will be relaunched with its new contract
- Responsibility for PPI will be built into new researchers’ contracts.

3. Support and Learning

**Progress**

- There is an identified budget for all PPI activity
- Finance is available for attendance at meetings, travelling and other expenses and care costs
- Generic training available free of charge through HCRW Support Centre. Bespoke briefing and training is available, identified at induction and reviews for theme RPs
- Mentor scheme is available to RPs with flexibility in its delivery in accordance with individual needs
- Briefings on PPI for researchers are promoted to and delivered on request with particular emphasis on early career researchers
- Information on PPI is promoted through engagement activities, the WCRC website, at conferences and through the HCRW Support Centre website
- Over the life of the WCRC contract with the Welsh Government PPI has become a ‘routine’ rather than ‘exception’ activity for centre staff, thanks to the collective leadership and example shown by managers at all levels of the organisation
- Support for PPI is coordinated by the Researcher and Lay leads with the support of a paid administrator.
- An audit of the skills and experience of current RPs has been undertaken to support their learning and to identify organisational gaps when recruiting additional RPs

**Future action**

- A review of the mentorship scheme is being undertaken to ensure it meets the needs of continuing and new RPs
- Further consideration will be given to payment of carer costs in the new contract
- One of the major priorities for the centre will be the further development and promotion of briefing and training opportunities in PPI for researchers, particularly to those at the beginning of their careers
- A review of the centre’s system recording for RPs’ activities will be undertaken to ensure they are appropriately recorded and recognised.

4. Communication

**Progress**

- The centre has a communications officer who coordinates communication activity through a website, twitter, Facebook, conferences and the centre’s paper publications. The centre produces an annual public facing report and has produced two reports focussed on PPI
- RPs are members of a range of Health and Social Care committees and locally and groups across the UK where they promote PPI to a wide community, for example the Wales School for Social Care Research, NIHR/INVOLVE and the PRIME Centre, based at Swansea and Cardiff Universities.
- A short summary of the centre’s PPI work was published in Problem Solving in Patient-Centred and Integrated Cancer Care [11]

**Future action**

- A review of the centre’s communication strategy will be undertaken for the new contract

5. Impact

**Progress**

- A diary based scheme for assessing the impact of RPs on research was trialled in two studies in 2017.
- The results were fed into a focussed project lasting 18 months, involving the Lay and Researcher Leads, two other RPs and the centre’s administrative officer. Its task was to produce a on impact assessment tool to be used initially within the centre
- The tool is based on a number of key principles: the clarity of the input to individual studies expected of RPs at the level of task, support and training for RPs and researchers in the use of the tool, emphasis on the mutuality of inputs expected of RPs by both parties, commitment to use of a single system of recording impact and reviewing at both within and at the end of the life of the study and a commitment to making the recording of impact both credible and manageable
- The rubric for the impact tool is set out in the SOP referred to in Table 7. A protocol of prompts to assist RPs and researcher when considering the tasks to be achieved by RPS is being developed.

**Future action**

- Publication of both the SOP and the protocol with training and support to RPs and researchers in the centre
- Roll out of the system to all new studies in the centre
- Review of the system after 12 months and improvements made
- Continuing input to any NIHR/INVOLVE working group looking at the impact of RPs on the quality of research

6. Governance

**Progress**

- Membership of RPs included at all levels of the centre’s governance structures from its Senior Leader’s Group (SLG) (Director, three Assistant Directors, Lay Lead for PPI and the operational manager) through to theme and work package advisory groups
- Early establishment of a PPI Group chaired by the Lay Lead to coordinate the development of PPI throughout the centre and to share practice and progress against agreed tasks
- Two RPs are recruited to each centre or trial study as recommended in its recruitment SOP
- Three of the theme RPs have helped to develop and draft a Cancer Research Strategy for Wales (CRest Cymru)
- Four of the theme RPs recruited to the Welsh Government’s Public Involvement Delivery Board to help further develop its approach to PPI
- The centre’s project plan for PPI which is reviewed on a weekly, then quarterly basis to help ensure progress continues to be made and reported to its funder and to its own SLG
- Theme RPs sit on a wide range of research and research governance groups across the UK to learn from good practice elsewhere and to disseminate the centre’s own practice
- A budget to fully fund the RPs and the administrative officer responsible for PPI in accordance with Welsh Government and INVOLVE guidelines
- Personal data is held and protected in accordance with GDPR

**Future action**

- Review governance arrangements for the centre in its new form for the next 5 years and ensure that RPs continue to be involved in its governance at all levels.

## Conclusion

The preceding paragraphs set out the WCRC’s approach to PPI and the progress it has made in developing its scheme of PPI in the last 5 years against:

- Its own set of KPIs and associated actions
- The broader UK Standards for Public Involvement.

Its progress in relation to implementing its scheme of PPI to date has been judged successful by its funder and its External Advisory Board. However, there is, and always will be, much to do, improvements to make.

In carrying its agenda forward the centre has in its favour:

- A strong commitment from its leaders, its researchers and RPs to PPI and, importantly, to working together.
- The review and planning processes it has put in place to underpin its work.
- The resources needed to fund its scheme, including money for administrative support
- Documentation to describe and support its processes from policy to review, including training and support and assessment of the impact on research of PPI
- A set of management structures within which PPI is embedded
- A commitment to continuous improvement.

Perhaps most importantly, all involved in the centre have given willingly and warmly of their time to make things happen. Together they have developed an organisational culture where PPI is the norm, a given. This is, perhaps the most important achievement of the last 5 years and gives confidence that the centre will continue to prioritise and improve its PPI.

## Data Availability

Not applicable.

## Abbreviations

PPI: Public and Patient Involvement
WCRC: Wales Cancer Research Centre
RP: Research Partner
